# Interface Effects in the Stability of 2D Silica, Silicide, and Silicene on Pt(111) and Rh(111)

**DOI:** 10.1021/acsami.4c05137

**Published:** 2024-05-15

**Authors:** Matthias Krinninger, Florian Kraushofer, Nils B. Refvik, Monika Blum, Barbara A. J. Lechner

**Affiliations:** † Functional Nanomaterials Group and Catalysis Research Center, Department of Chemistry, TUM School of Natural Sciences, Technical University of Munich, Lichtenbergstr. 4, 85748 Garching, Germany; ‡ Department of Physics, University of Alberta, Edmonton, Alberta T6G 2E1, Canada; § Chemical Sciences Division, Lawrence Berkeley National Laboratory, Berkeley, California 94720, United States; ∥ Advanced Light Source, Lawrence Berkeley National Laboratory, Berkeley, California 94720, United States; ⊥ Institute for Advanced Study, Technical University of Munich, 85748 Garching, Germany

**Keywords:** silica thin films, silicide, model catalyst support, in situ, STM, XPS

## Abstract

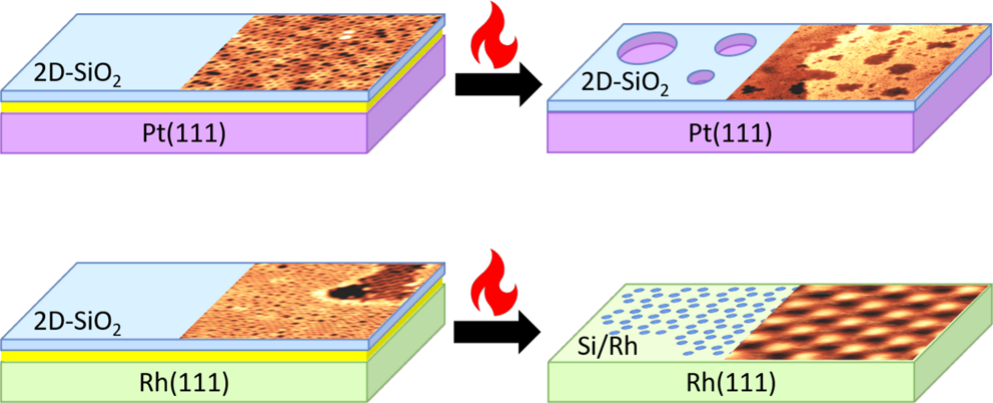

Ultrathin two-dimensional silica films have been suggested as highly defined conductive models for fundamental studies on silica-supported catalyst particles. Key requirements in this context are closed silica films that isolate the gas phase from the underlying metal substrate and stability under reaction conditions. Here, we present silica bilayer films grown on Pt(111) and Rh(111) and characterize them by scanning tunneling microscopy and X-ray photoelectron spectroscopy. We provide the first report of silica bilayer films on Rh(111) and have further successfully prepared fully closed films on Pt(111). Interestingly, surface and interface silicide phases play a decisive role in both cases: On platinum, closed films can be stabilized only when silicon is deposited in excess, which results in an interfacial silicide or silicate layer. We show that these silica films can also be grown directly from a surface silicide phase. In the case of rhodium, the silica phase is less stable and can be reduced to a silicide in reductive environments. Though similar in appearance to the “silicene” phases that have been controversially discussed on Ag(111), we conclude that an interpretation of the phase as a surface silicide is more consistent with our data. Finally, we show that the silica film on platinum is stable in 0.8 mbar CO but unstable at elevated temperatures. We thus conclude that these systems are only suitable as model catalyst supports to a limited extent.

## Introduction

Silica is a prototypical catalyst support material due to its high stability and low cost. To gain an atomic-scale understanding of catalytic processes, surface science experiments have traditionally been applied on simplified model catalysts.^1^ Often, these studies—aiming to explore specific properties of supported catalyst particles in, e.g., ethylene hydrogenation^2,3^ or CO oxidation^4^ reactions—use relatively thick silicon dioxide (SiO_2_) films of several nanometers.^5^ However, the choice of experimental techniques is then limited by the extremely low conductivity of silica, and in particular, thick films are unsuitable for surface microscopy due to their inherent roughness. Hence, ultrathin two-dimensional (2D) silica films have been suggested as highly defined, conductive, and atomically flat models for fundamental studies on silica-supported catalysts.^6,7^ To avoid side reactions, closed films that fully cover the underlying metal substrate are of key importance.

The structure of these films has been extensively studied over the last two decades, starting on Mo(112)^8^ as an underlying substrate, with the first scanning tunneling microscopy (STM) images of its atomically resolved structure reported in 2005.^9^ Here, a single-layer, 2D network could be grown that consists of highly ordered [SiO_4_] tetrahedra as building blocks of which one oxygen atom is bound to a Mo atom of the surface. Later, a bilayer structure was found on Ru(0001) and termed “2D silica”.^10,11^ Because the Ru–O bond is weaker than the Mo–O one, none of the oxygen atoms of the building blocks are bound to the surface. Instead, they form two planes of corner-sharing tetrahedra with one oxygen bridging these two layers, while the bonding forces between the film and the underlying metal are only van der Waals interactions. These bilayers can be further categorized into crystalline, where the building blocks form exclusively six-membered rings and amorphous/vitreous films, with varying ring sizes of four to nine. Crystalline and amorphous films could be obtained selectively by tuning the cooling rate after the oxidative annealing step during the synthesis.^12^ Over the years, 2D silica has also been reported on a variety of other substrates, including Pd(100),^13^ Pd(111),^14,15^ a Ni_*x*_Pd_1–*x*_(111) alloy film,^16^ Ru and Co nanoparticles,^17^ Cu-supported graphene,^18,19^ and Pt(111).^20^

The silica films exhibit high thermal stability, as shown on Mo(112) and Ru(0001).^21,22^ On these substrates, “O-poor” and “O-rich” films could be prepared by annealing in UHV or in an oxygen atmosphere. The difference between these two films lies in the chemisorption of O at the surface under the film. While it is possible to convert “O-poor” films into “O-rich” films through oxygen annealing in the case of Mo(112), the UHV-annealing-based pathway in the opposite direction is blocked due to the high binding energy of oxygen to the Mo(112) surface.^21^ In contrast, the transformation from one phase to the other is fully reversible on Ru(0001). This is reflected in X-ray photoelectron spectroscopy (XPS), where both the Si 2p and the O 1s peaks shift to higher binding energies by up to 0.8 eV after UHV annealing, depending on the temperature.^22^

Under oxygen-poor conditions, these silica phases are in competition with surface silicides, which have also been observed on most of the relevant metals. On Pt(111), surface segregation of Si impurities was reported to result in a (√19 × √19)*R*23.4° structure under high-temperature annealing.^23^ A more systematic study by Nashner et al. used vapor-deposited SiH_4_ to show that a variety of different surface silicide structures can be formed depending on the Si/Pt ratio at the surface.^24^ In some cases, these silicon-rich phases have also been interpreted as a “silicene” with a structure akin to graphene: Feng et al. observed novel structures of Si on Ag(111) in STM and attributed them to different silicene phases.^25^ Their model consists of hexagonally arranged, buckled six-rings that form a (4 × 4) reconstruction with respect to the underlying Ag(111). However, Švec et al. later proposed a model for the (√19 × √19)*R*23.4° silicide on Pt(111) and convincingly argued that the “silicene” phase on Ag(111) has the same appearance in STM and can likely be explained in the same way.^26^ The model by Švec et al. consists of Si atoms incorporated into the Pt lattice, forming two PtSi_3_ tetramers, each with a central Pt atom per unit cell. One tetramer sits on top of a Pt atom in the second layer, while the other one sits above a hollow site, resulting in the hexagonally arranged protrusions and depressions observed in STM images.

In the case of Pt(111), no fully closed 2D silica film has been reported to date. The oxygen affinity of the metal was found to be the decisive criterion for the film structure, where substrates with high heats of dissociative oxygen adsorption favor crystalline monolayer films, while a lower heat of adsorption favors vitreous bilayers; only a minor influence was attributed to the lattice match.^20^ Hence, we chose Rh as an additional substrate since its heat of dissociative oxygen adsorption (−182 kJ mol^–1^) lies between the values for Ru (−220 kJ mol^–1^) and Pt (−133 kJ mol^–1^).^27^ Additionally, the 2.69 Å lattice constant of Rh(111)^28^ results in a better (factor-of-two) lattice match with the calculated periodicity of a free-standing, crystalline SiO_2_ bilayer structure (5.30 Å)^13,29^ than is obtained for Ru(0001) (2.71 Å)^30^ or Pt(111) (2.77 Å).^31^ Altman et al.^32^ recently stated that the lattice mismatch and oxygen affinity of Rh(111) do not follow the same trend as other investigated metal substrates, thus making it highly interesting to study in order to disentangle these two influencing factors.

Here, we use STM, XPS, and low energy electron diffraction (LEED) to investigate the growth of 2D silica on Pt(111) and Rh(111), characterize the different resulting structures on these metals, and study the stability of the films under reducing conditions in CO at near-ambient pressure (NAP) as well as at high temperatures in ultrahigh vacuum (UHV). Furthermore, we discuss the possible influence of an intermixing of Si with the metal substrate at the 2D silica/Pt(111) interface on the film structure.

## Results

**Figure 1:**
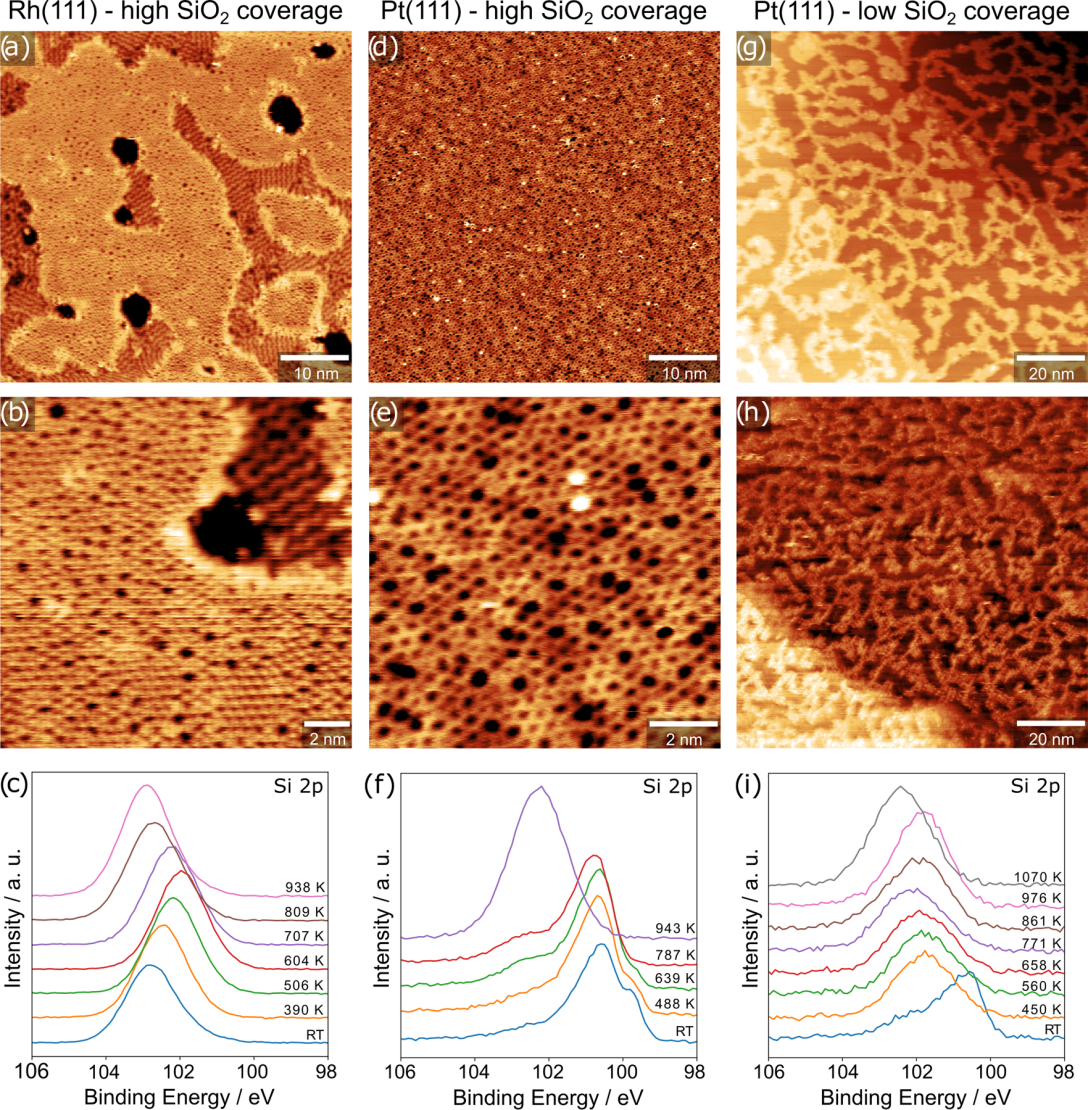
STM images of 2D silica and corresponding XPS Si 2p spectra acquired during film synthesis in 5.0 × 10^–6^ mbar O_2_ for (a–c) a full monolayer on Rh(111), (d–f) a full monolayer on Pt(111) and (g–i) a submonolayer film on Pt(111), annealed at different temperatures. STM images were acquired in UHV at room temperature, with the following tunneling parameters: (a) *I*_t_ = 0.12 nA, *V*_b_ = 0.77 V, (b) *I*_t_ = 0.10 nA, *V*_b_ = 0.77 V, (d) *I*_t_ = 0.34 nA, *V*_b_ = 0.36 V, (e) *I*_t_ = 0.34 nA, *V*_b_ = 0.36 V, (g) *I*_t_ = 0.42 nA, *V*_b_ = 0.33 V, and (h) *I*_t_ = 0.65 nA, *V*_b_ = 4.51 V. XPS parameters: excitation energy of 300 eV.

First, we look at the preparation of 2D silica on Rh(111). We prepared the film by deposition of 6 Å Si (2 ML Si) in an oxygen atmosphere of 1.5 × 10^–6^ mbar O_2_, holding the Rh(111) crystal at 165 K. Subsequently, we annealed the sample at 1200 K in 5.0 × 10^–6^ mbar O_2_ for 5 min. Figure 1a shows an STM image of the resulting silica film with a nearly complete coverage of the sample with 2D silica of different morphologies coexisting and the presence of holes. Note that we do not find any significant bias-dependent contrast changes. Higher magnification of the same sample (Figure 1b) reveals that the majority of the surface is covered with a vitreous film that also shows crystalline domains (hexagonal pattern) and coexists with a distinct “zigzag” structure (appearing as lower-lying) that also has been reported for Ru(0001) and Pt(111).^33,34^ We also observed this slightly oxygen-enriched zigzag polymorph (SiO_2.17_ compared to SiO_2_ for bilayer silica) in smaller quantities on Pt(111) (see Figure S1, Supporting Information) but only for annealing times *t* ≥ 30 min in 5.0 × 10^–6^ mbar oxygen. We attribute the more facile formation on Rh to its higher oxygen affinity compared to Pt. Monitoring the growth of silica on Rh(111) via XPS indicates a complex growth mechanism, with the underlying Rh(111) being more or less oxidized depending on the temperature. After deposition, the Si 2p signal has a peak at a binding energy of 102.8 eV (Figure 1c), likely due to oxidation during the initial evaporation process or at the Rh(111) surface, which is known to adsorb oxygen at room temperature.^35−37^ Monitoring the signal in situ during annealing first shows a shift to lower binding energies up to 604 K before shifting back to higher binding energies at even higher temperatures. The O 1s spectra (Figure S2a, Supporting Information) show the same shifting behavior at the corresponding temperatures (Table S1, Supporting Information) and exhibit an additional shoulder at lower binding energies that evolves proportional to the energy shift, i.e., the lower the binding energy of the O 1s and Si 2p peaks, the more intense the shoulder. This is in good agreement with a previous study of 2D silica on Ru(0001), in which this low binding energy shoulder was assigned to interfacial oxygen adsorbed at the Ru(0001) surface, which correlates with lower binding energies of both the Si 2p and the O 1s signals.^22^ Correspondingly, we interpret our results as showing the different amounts of interfacial oxygen absorbed on Rh(111) during the film formation. For the final film annealed to a temperature of 938 K, the Si 2p signal is at 102.9 eV with a full width at half-maximum (FWHM) of ∼1.65 eV, which is in good agreement with reported binding energy values for Pt(111)^20^ (102.8 eV) and Ru(0001)^10,22^ (102.5 eV).

When changing the substrate from Rh(111) to Pt(111) while keeping the synthesis procedure for the 2D silica the same, the film appears much more homogeneous in the STM (see Figure 1d,e), exhibiting only the vitreous bilayer as reported by Yu et al.^20^ An amorphous network of different pore sizes spans the surface and shows a slight corrugation, as well as very few defects appearing as bright blobs, which we interpret as third layer species. However, in contrast to the previous reports, we could achieve a film that is free of holes across wide terraces, which we attribute to the deposition of silicon in excess and short oxygen annealing time. For longer annealing times in the same atmosphere, the resulting film is structurally similar, but some holes appear (see Figure S3b,c, Supporting Information).

Further insight into the growth and subsequent destabilization of the film is provided by XPS data acquired during annealing (see Figures 1f and S2b). In the Si 2p spectrum after deposition, the main signal appears at 100.6 eV, together with a relatively strong shoulder at 99.8 eV and a small peak at around 102.4 eV. These species are not well-defined after deposition at room temperature but likely correspond to silica species that are only weakly oxidized on the preoxidized Pt surface and/or due to interaction with gas-phase oxygen. Upon heating to 787 K, the main signal shifts by about 0.2 eV to higher binding energy, the low binding energy shoulder decreases in intensity, and the high binding energy species increases. At 943 K, all Si species previously at 100.6 eV and below were converted to the one at 102.4 eV with a FWHM of ∼1.8 eV, which we interpret as the entire film being fully oxidized.

To gain a deeper understanding of the growth mechanism of 2D silica on Pt(111), we also deposited 1/3 of the amount used in the previous syntheses and chose a lower annealing temperature of 850 K. The resulting silica (Figure 1g) shows no large islands but rather a dendritic network across several terraces, suggesting an even spreading already at lower temperatures. In this case, additional annealing of 90 min at 850 K, 35 min at 1000 K, and 5 min at 1200 K in the synthesis atmosphere does not change the topography (Figure 1h). In the initial XPS spectrum after deposition (blue line in Figure 1i), the signal is similar in binding energy (main peak ∼100.6 eV) to the one observed for the full surface covering 2D silica. However, at this lower coverage, the signal shifts already at 450 K to a higher binding energy of ∼101.8 eV and appears as a single peak. Finally, at 1070 K, the peak appears at a binding energy of 102.4 eV with a FWHM of again ∼1.8 eV. Thus, the final chemical state after prolonged annealing is the same as for higher coverage silica films. It is interesting to note, however, that before this final data point (obtained by prolonged high-temperature annealing up to 976 K), the main Si 2p component observed for the submonolayer film is found at significantly higher binding energy (∼101.8 eV) than the highest Si 2p peak of the closed film (100.6 eV) and instead appears closer to the shoulder at 102.4 eV. Considering that the shift between 976 K (pink line in Figure 1i) and 1070 K (gray line in Figure 1i) is likely caused by the desorption of interfacial oxygen,^22^ this might indicate that full oxidation is achieved at much lower temperatures for the low Si coverage (see also O 1s spectra in Figure S2c, Supporting Information). As discussed below, we interpret this coexistence of multiple Si 2p components throughout the annealing process as evidence for a platinum silicide or silicate phase that is present below the silica bilayer when silicon is deposited in excess.

Since the measurements were performed in different setups, the STM images do not directly correspond to the XPS spectra in Figure 1, and annealing times during film synthesis differed somewhat due to the signal monitoring in XPS. To address this discrepancy, we also performed Auger electron spectroscopy (AES) of the fully closed 2D silica directly after the STM measurements (Figure S3a, Supporting Information). With a kinetic energy of ∼82 eV, the main Si peak of the closed film shown in Figure 1d,e (orange line in Figure S3a) is located between literature values for elemental silicon (92 eV) and bulk silicon dioxide (76 eV).^38^ As mentioned above, further annealing steps at 1200 K (green line) and 1300 K (red line) in 5.0 × 10^–6^ mbar, as well as 1000 K in UHV (purple line), caused holes to appear in the film (Figure S3b,c), while the AES signal shifts to lower kinetic energies, in agreement with the XPS results.

**Figure 2:**
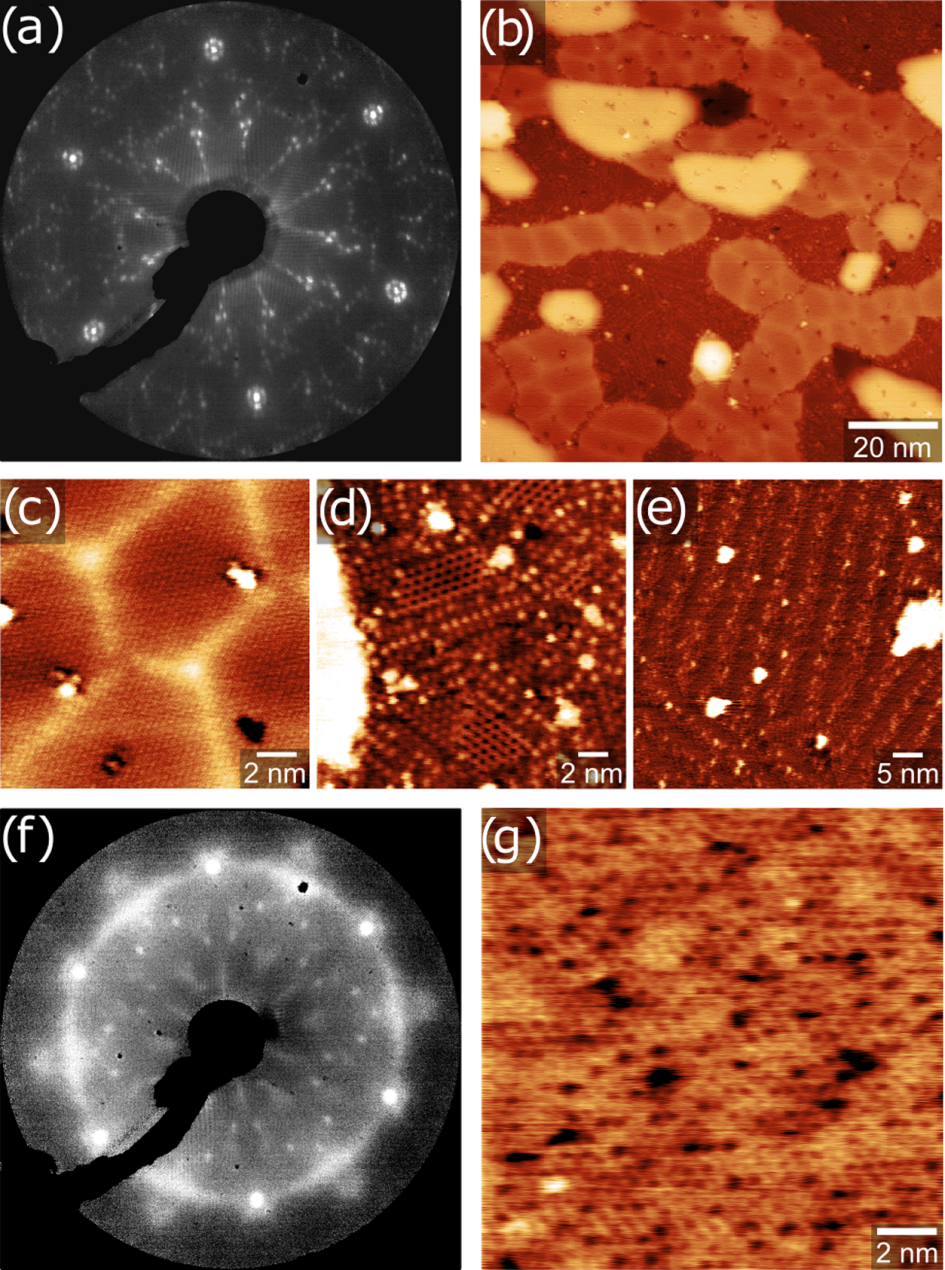
(a) LEED and (b–e) STM images of a Pt surface silicide and (f, g) respective data for the resulting silica film after oxidation. STM images were acquired in UHV at room temperature, with the following tunneling parameters: (b) *I*_t_ = 0.51 nA, *V*_b_ = 0.88 V, 100 × 100 nm^2^, (c) *I*_t_ = 0.47 nA, *V*_b_ = 0.79 V, 15 × 15 nm^2^, (d) *I*_t_ = 0.47 nA, *V*_b_ = 0.79 V, 20 × 20 nm^2^, (e) *I*_t_ = 1.33 nA, *V*_b_ = 0.84 V, 50 × 50 nm^2^, and (g) *I*_t_ = 0.39 nA, *V*_b_ = 0.35 V, 15 × 15 nm^2^. LEED energies: (a) 110 eV and (f) 120 eV.

To elucidate the nature of a potential interfacial silicide and to investigate whether 2D silica can be formed via direct oxidation of such a phase, we deposited the same Si amount as used to obtain the fully closed 2D silica on bare Pt(111) and annealed in UHV (instead of in an oxygen atmosphere) at 800 K for 10 min. In the STM, we can observe a variety of different coexisting surface structures (Figure 2b–e) that are also reflected in the complexity of the resulting LEED pattern (Figure 2a). The latter one appears very similar to the LEED images for a Si/Pt ratio of 0.43 and 0.30 reported by Nashner et al., which were formed when transitioning from a (√7 × √7)*R*19.1° to the (√19 × √19)*R*23.4° surface silicide.^24^ With additional annealing in 5.0 × 10^–6^ mbar oxygen at 1200 K for 5 min, we again obtained a 2D silica film with the same appearance in STM (Figure 2g) as for the synthesis starting from bare Pt(111). However, a clear difference is seen in LEED: When 2D silica is grown by immediately annealing in oxygen after deposition (as in Figure 1d,e), the LEED pattern (Figure S4, Supporting Information) shows only the spots corresponding to the hexagonal Pt(111) substrate and a diffuse ring, indicating different ring sizes and orientations in the 2D silica. In contrast, when the silica film is grown from the silicide phase, LEED shows numerous additional spots, indicating an ordered superstructure (Figure 2f). Since the silica layer observed in STM (Figure 2g) shows no particular ordering, these superstructure spots must reflect a subsurface phase, which indicates that the silicide is still present underneath the SiO_2_ bilayer. As the same amount of silicon was deposited in both cases, we can assume that an interfacial silicide is also present underneath the films seen in Figure 1d,e but was not ordered sufficiently to be visible in LEED.

**Figure 3:**
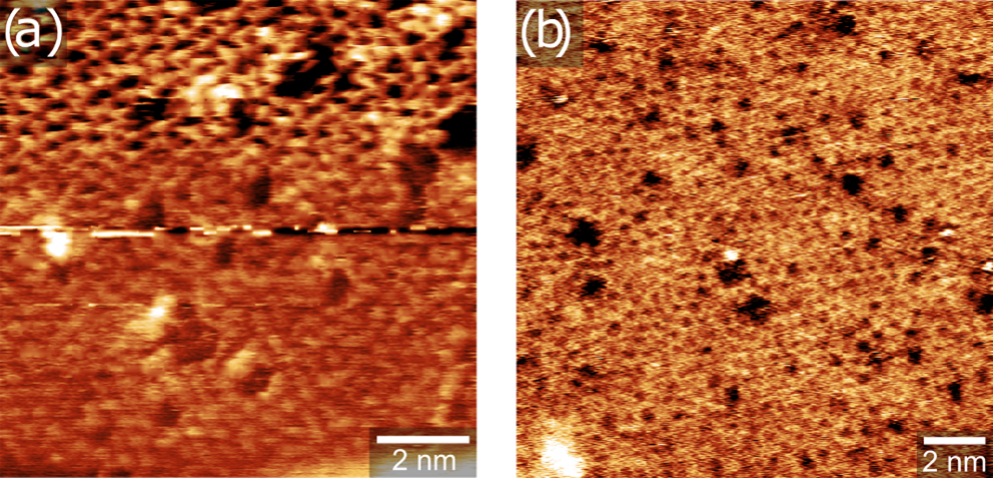
Room temperature STM images of 2D silica on Pt(111) (a) in 0.8 mbar CO and (b) under UHV after pumping back down. Tunneling parameters: (a) *I*_t_ = 0.86 nA, *V*_b_ = 0.50 V, 10 × 10 nm^2^ and (b) *I*_t_ = 0.34 nA, *V*_b_ = 0.32 V, 15 × 15 nm^2^.

In order to see if the 2D silica on Pt(111) is suitable as a support material for model catalysis studies under NAP conditions, we conducted STM measurements in a CO atmosphere at room temperature. An in situ NAP-STM image under 0.8 mbar CO is shown in Figure 3a. The film was prepared in the same manner as the one shown in Figure 1d,e. While the contrast is somewhat blurred in the CO atmosphere compared to UHV (Figure 1e) possibly due to interaction of the STM tip with the gas phase, the vitreous SiO_2_ network is still clearly visible. Figure 3b shows an image of the same sample that had been exposed to the CO atmosphere for 2.5 h and pumped back down to UHV. Again, no major changes are apparent with respect to the as-prepared film.

**Figure 4:**
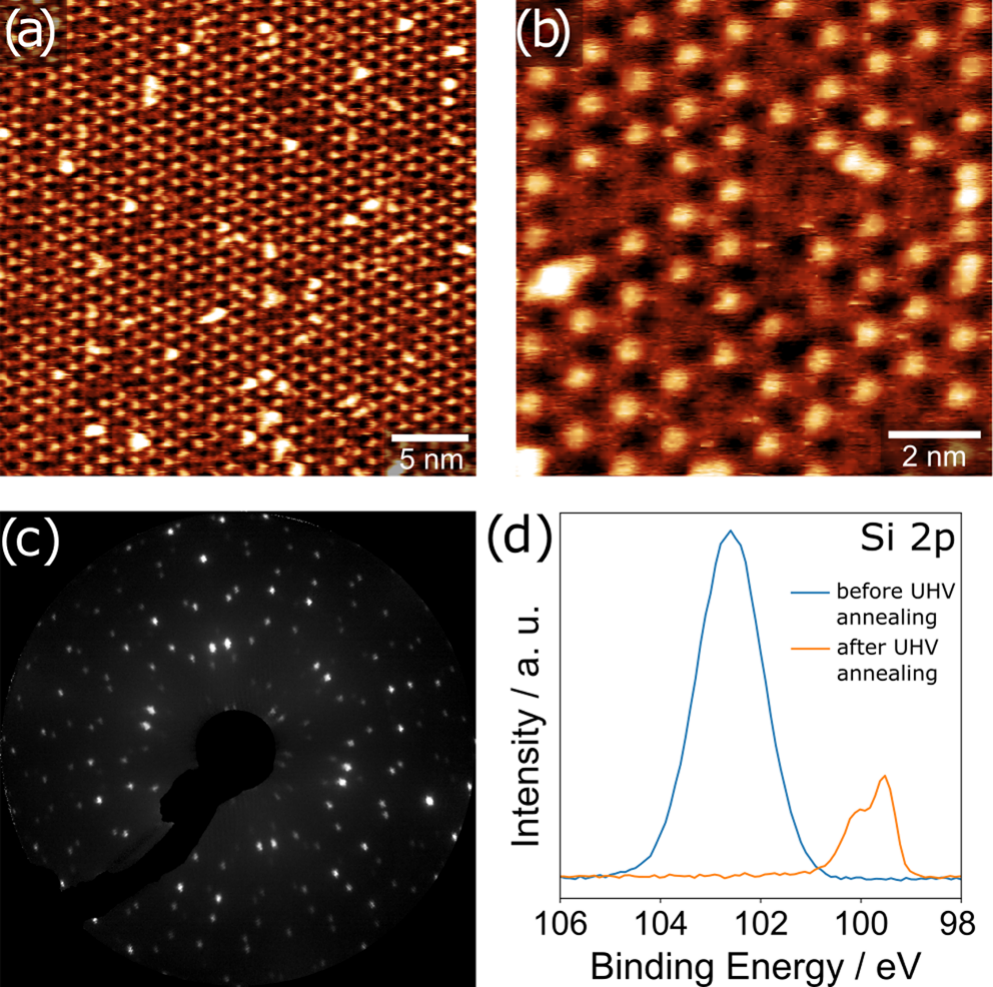
STM images, LEED, and XPS spectra of reduced silica on Rh(111). (a) STM image of the Rh surface silicide resulting from the reduction of 2D silica on Rh(111) via UHV annealing at 1200 K for 30 min. (b) Higher magnification STM image of the same surface. (c) LEED image (103 eV) of the surface silicide exhibiting a (√19 × √19)*R*23.4° superstructure with respect to Rh(111). (d) Si 2p region of 2D silica (blue, before UHV annealing) and the surface silicide (orange, after UHV annealing at 946 K). Tunneling parameters: (a) *I*_t_ = 0.22 nA, *V*_b_ = 2.12 V, 30 × 30 nm^2^ and (b) *I*_t_ = 0.12 nA, *V*_b_ = 1.27 V, 10 × 10 nm^2^. XPS parameters: excitation energy of 300 eV.

Further to being stable in gas atmospheres, an inert model catalyst support should also be stable at elevated temperatures. As mentioned, a shift in binding energy of the Si 2p signal of up to 0.8 eV occurred when converting “O-rich” into “O-poor” 2D silica on Ru(0001) at annealing temperatures of up to 1150 K.^22^ Similarly, we observed only a very small shift of 0.1 eV upon annealing the full coverage film on Pt(111) at 960 K (Figure S5, Supporting Information). However, a drastically different behavior is observed on Rh(111). Upon annealing at 946 K, the Si 2p signal appeared as a sharp doublet at 99.5 and 100.1 eV with a significant decrease in total signal intensity (Figure 4d). This binding energy corresponds well to different bulk Rh silicides in the literature (99.5–99.7 eV),^39,43^ as well as surface PtSi.^40^ Additionally, the O 1s signal is completely lost (Figure S6, Supporting Information). STM images acquired after annealing an SiO_2_ film like the one shown in Figure 1a,b in UHV at 1200 K for 30 min are shown in Figure 4a,b. There are no traces of any of the 2D silica phases left, and instead, we observe an ordered hexagonal structure of protrusions with a lattice constant of ∼11 Å. The corresponding LEED image (Figure 4c) shows the same (√19 × √19)*R*23.4° reconstruction as reported for surface-segregated Si on Pt(111).^23^ Overall, both STM and LEED images, as well as the binding energy of the Si 2p peak, are consistent with surface silicides reported on Pt(111)^23,26^ and could be explained with the model proposed by Švec et al.^26^

**Figure 5:**
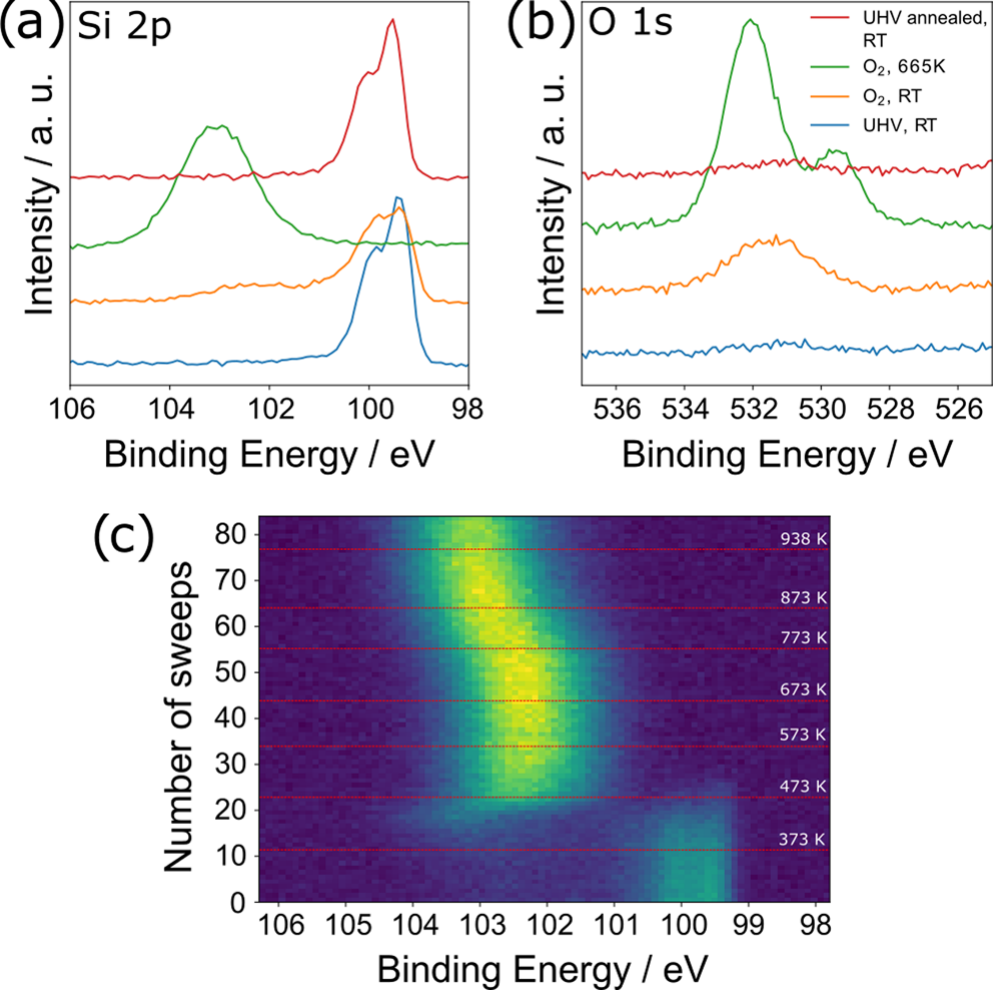
XPS spectra of the oxidation of the Rh surface silicide. (a, b) Si 2p (excitation energy 300 eV) and O 1s (excitation energy 650 eV) spectra of the surface silicide directly after 2D silica reduction (blue), partial oxidation after dosing 5 × 10^–6^ mbar oxygen (orange), fully reoxidized silica after heating to 938 K in oxygen (green), and surface silicide after renewed reduction at 938 K in UHV and cooling to RT (red). (c) Time series Si 2p spectrum of the oxidation of the Rh surface silicide, beginning at RT in 5 × 10^–6^ mbar oxygen. Sweep *N* = 0 corresponds to the orange line, and *N* = 84 to the green line in (a, b); each sweep takes 46 s. 100 K temperature steps and the maximum temperature reached are indicated by red lines.

To test if the reduction of 2D silica on Rh(111) is reversible, we heated the surface silicide again in an oxygen atmosphere at 5 × 10^–6^ mbar. In the resulting XPS spectra (Figure 5a,b), an immediate change occurs after O_2_ exposure already at RT. Comparing the Si 2p spectrum before exposure (blue line in Figure 5a) with the one after exposure (orange line), the silicide signal decreases and an additional small signal appears at a binding energy of ∼102.2 eV. Simultaneously, an O 1s signal appears at 531.4 eV. It seems likely that these signals are caused by oxygen coordinating or binding to the Si atoms on the surface. Subsequently, we monitored the oxidation of the surface silicide by continuously measuring the Si 2p signal while heating (Figure 5c). Starting at sweep 9, which corresponds to a temperature of ∼345 K, the silicide signal progressively decreases while the signal at higher binding energy increases. At about sweep 30 (534 K), the silicide signal is completely gone, and the intensity of the high binding energy signal is fully developed. Further heating causes only a small shift to higher binding energy due to the partial desorption of oxygen from Rh below the SiO_2_ film, consistent with the spectra in Figure 1c. The green lines in Figure 5a,b show high-resolution spectra of Si 2p and O 1s, respectively, at the final temperature of 665 K. The Si 2p signal again appears at the binding energy of the fully oxidized Si at 102.9 eV. The O 1s signal appears to be split into two peaks, the one at higher binding energy corresponding to Si–O and the one at lower binding energy corresponding to Rh–O.^22^ However, the much higher signal intensity observed before initial reduction (blue curve in Figure 4d) is clearly not recovered by the reoxidation.

Finally, we tested how fast the reduction of the film occurs by closing the oxygen valve at 665 K while monitoring the Si 2p signal (Figure S7, Supporting Information). Within ∼5 min (7 sweeps, 46 s each), the silica is again completely transformed to a silicide. The Si 2p and O 1s high-resolution spectra after cooling down are shown in Figure 5a,b (red curves).

## Discussion

Our STM results demonstrate the successful formation of a fully closed 2D silica film on Pt(111). We attribute this achievement to the brief oxygen annealing period of only 5 min following silicon deposition, coupled with the use of silicon in excess. There are multiple indications that this excess Si is still present below our silica film: First, when comparing XPS data of high and low Si loading (Figure 1f,i), the submonolayer film shows only one component in the Si 2p region while multiple peaks are found for the closed films. Notably, when depositing Si in excess, full oxidation is only achieved after prolonged annealing at high temperatures, which in the STM always leads to the formation of holes in the film. We have also shown that a silica film can be grown directly from a surface silicide, and LEED (Figure 2f) shows that an ordered phase persists below the film under these conditions. This is likely the ordered silicide (Si_*x*_Pt), although XPS suggests at least partial oxidation to an interfacial silicate (Si_*x*_O_*y*_Pt). Interestingly, no superstructure spots are found in LEED of directly synthesized films (Figure S4). It is possible that when annealing is performed in oxygen directly, oxidic precursor states of the silica film prevent the surface silicide from ordering to the same degree as when UHV annealing is performed (Figure 2a–e).

We propose that this interface layer functions as a crucial “buffer layer”, which is the decisive factor in stabilizing the 2D silica, preventing the formation of any undesirable holes in the film. The resulting 2D silica structure is stable in a CO atmosphere of up to 0.8 mbar. However, the silicide/silicate buffer layer is unstable when subjected to high temperatures (>1000 K) in UHV or oxygen, which we attribute to its reductive or oxidative degradation, respectively. It seems plausible that during reductive degradation, the surplus silicon initially forms a surface silicide up to a certain threshold but ultimately undergoes diffusion into the Pt bulk, given silicon’s notable mobility in this temperature range.^23,41,42^ On the contrary, if the interface layer undergoes further oxidation, there is the possibility of additional silicon being incorporated into the 2D silica. This incorporation may result in subtle surface corrugations due to minor buckling, potentially culminating in the out-of-plane growth of silica, as indicated by the increased roughness observed when attempting to image the surface.

Switching the underlying substrate to Rh(111), it is evident that 2D silica on Rh(111) aligns with the crystallinity trend concerning the heat of dissociative oxygen adsorption, positioned between Ru(0001)—where both crystalline films and zigzag structures are viable—and Pt(111), where 2D silica exclusively adopts an amorphous structure. In this regard, Rh(111) behaves close to Ru(0001), as at least some crystalline areas can be observed in Figure 1a,b, and better ordering could likely be achieved by tuning the annealing temperature and cooling ramp. However, unlike on Ru or Pt, the thermal reaction from 2D silica to a surface alloy/silicide is extremely facile on Rh, which destabilizes the silica film despite the better lattice match.

Unlike earlier studies on 2D silica on Ru(0001), we were not able to find a preparation that yields a fully closed silica film on Rh(111). In this respect, Rh is more similar to previous studies on Pt(111) but with the difference that the formation of a stabilizing buffer layer is precluded. This limitation seems to arise from the prompt oxidation of the deposited Si on the Rh(111) surface, as deduced from the XPS data (Figure 1c). Also unlike Ru and Pt, full reduction of the 2D silica is possible on Rh(111) by relatively mild UHV annealing, resulting in the formation of a (√19 × √19)*R*23.4° surface silicide like the one reported on Pt(111).^23,26^ In a similar vein, Labich et al. reported the reaction of Rh clusters deposited on a 90 Å thick SiO_2_ film on Mo-foil, forming the rhodium silicide Rh_3_Si upon heating to 873 K in UHV, emerging at a binding energy of 100.3 eV in XPS.^43^ Because of the much higher heat of formation of SiO_2_ compared to the metal silicide, the proposed reaction mechanism occurs via oxygen transfer to Rh, followed by thermal desorption of oxygen, rather than via the formation of the thermodynamically less favorable metal oxide. Therefore, we ascribe rhodium’s potential to cleave Si–O bonds, coupled with oxygen’s complete desorption from the surface as evidenced in XPS (Figures 4d and S6), as the key factors driving the formation of the pure (√19 × √19)*R*23.4° surface silicide.

This reduction and the subsequent reoxidation of the surface silicide on Rh(111) are chemically reversible, but the large signal loss resulting from the initial reduction is irreversible. This is likely due to the diffusion of Si into the bulk, which is known to be fast in this temperature regime as the two elements are miscible.^44,45^ Volatile SiO species that can then desorb from the surface may also form during film degradation, as shown for native silica.^46−48^ Note that we also tried to measure STM of the resulting reoxidized silica which turned out to be challenging, indicating that the structure is rather growing three-dimensionally instead of forming flat islands of lower coverage.

Finally, it is interesting to reiterate at this point that the (√19 × √19)*R*23.4° silicide on Rh(111) seems virtually identical to the one on Pt(111), and that similar STM images have previously been interpreted as silicene phases on Ag(111).^25^ Fundamentally, such a decoupled phase seems chemically unlikely on Pt and even more so on Rh, where silicide formation is facile. As noted already in the Introduction section, an extensive and convincing argument against a silicene phase on Pt(111) has already been presented by Švec et al.^26^ More recently, a study by Küchle et al. has presented evidence that even on Ag(111), where the topmost layer does seem to consist of a 2D arrangement of silicon atoms, this arrangement is supported on an Ag/Si layer acting as a buffer layer.^49^ While the chemistry of the Pt silicide stabilizing a closed silica film is clearly quite different, this nicely highlights the importance that mixed interfacial layers may have on the stability of thin films at surfaces.

## Conclusions

We have shown that fully closed 2D silica films can be obtained on Pt(111) when they are stabilized by an interfacial silicide or silicate buffer layer. The films can also be directly synthesized from a surface silicide precursor and show high stability against relatively high pressures of CO. However, the stabilization remains challenging for both oxidative and reductive conditions at elevated temperatures. While the closed film is thus not stable enough to serve as a support in model catalysis investigations at near-ambient pressures and elevated temperatures, the film with holes exhibits high stability, in line with the reports for Ru(0001) and Pt(111).

On Rh(111), we have obtained films with mixed morphologies, specifically vitreous, crystalline, and “zigzag” structures, as reported previously on other substrates. In stark contrast to previously investigated metal substrates, however, 2D silica on Rh(111) can be fully reduced by UHV annealing, forming a surface silicide with a (√19 × √19)*R*23.4° periodicity. While recovery of the films is hampered by the fact that a large portion of the Si is lost, either to the Rh bulk or to the gas phase as SiO, the initial reduction is chemically fully reversible.

## Experimental Section

Pt(111) single crystal samples (from SPL for STM and from Mateck for XPS measurements) were prepared by several cycles of sputtering (Ar^+^, 5 × 10^–5^ mbar, 1.0 keV, 15 min) and annealing (1200 K, 1 min, followed by 800 K, 15 min, both in *p*(O_2_) = 1 × 10^–7^ mbar). Rh(111) single crystal samples (from SPL) were prepared by several cycles of sputtering (Ar^+^, 3 × 10^–5^ mbar, 1.0 keV, 15 min) and annealing (1200 K, 15 min in *p*(O_2_) = 5 × 10^–7^ mbar with the last cycle in UHV). The samples were heated using an e-beam heater. The temperature was measured with a type K thermocouple attached to the backside of the crystal.

For the STM and LEED experiments, Si deposition was carried out with an EBE-1 evaporator by SPECS with a Si rod from Goodfellow (diameter: 2.0 mm, purity: 99.999%, crystalline), monitored with a quartz crystal microbalance by OmniVac. In the deposition procedure, we monitored the signal of the microbalance in an oxygen atmosphere until a stable deposition rate was reached. Then, the microbalance was removed, and the sample was placed in the same position to deposit the desired amount of silicon. Note that we used the density of elemental silicon for the rate monitoring, which could result in a systematic error, considering that the deposit is at least partially oxidized Si. STM measurements were performed with an SPM Aarhus 150 NAP-STM by SPECS in constant current mode with an electrochemically etched tungsten tip. Bias voltages (*V*_b_) refer to the sample voltage with respect to the tip. Image correction was carried out with the SPM software Gwyddion using the plane correction and row alignment tools.^50^ LEED images were acquired with an ErLEED 150 by SPECS. All AES measurements were performed with a DESA 150 by Staib Instruments with a primary electron energy of 5 keV.

All XPS measurements were conducted at beamline 9.3.2 of the Advanced Light Source at the Lawrence Berkeley National Laboratory. Here, the same Si rod was used for deposition with an EBE-4 evaporator by SPECS. All O 1s spectra were measured with a beam energy of 650 eV and were referenced to the binding energy of the Pt 4f or Rh 3d peak, respectively. The Si 2p spectra were obtained with a beam energy of 300 eV and referenced to the binding energy of the Pt 4f or Rh 4p peak, giving us a similar surface sensitivity for both O 1s and Si 2p core levels. The energy resolution was ∼0.2 eV throughout the available energy range. The spot size was ∼1 mm^2^ on the sample, i.e., much larger than the area measured in STM. Probing different areas of the sample with AES, LEED, and XPS yielded perfectly reproducible results, indicating that the sample is homogeneously covered. For heating, a pyrolytic boron nitride heater was used; the temperature was monitored with a type K thermocouple at the front-side of the single crystal.

## Supporting Information Available

Additional figures: STM images showing the SiO_2.17_ zigzag phase; O 1s spectra acquired during SiO_2_ film synthesis; AES spectra of the SiO_2_ film on Pt(111) as prepared and after different annealing steps with corresponding STM images; LEED image of the SiO_2_ film on Pt(111); Si 2p XPS spectra of the SiO_2_ film on Pt(111) before and after annealing in UHV; O 1s XPS spectra of the SiO_2_ film on Rh(111) before and after annealing in UHV; Si 2p XPS spectra of the SiO_2_ film reduction on Rh(111); and table listing binding energies of Si 2p and O 1s during SiO_2_ film synthesis at different temperatures (PDF)

## Abbreviations

STM: scanning tunneling microscopy
XPS: X-ray photoelectron spectroscopy
LEED: low energy electron diffraction
UHV: ultrahigh vacuum
NAP: near-ambient pressure
FWHM: full width at half-maximum
