# Supplementary material for: Interface Effects in the Stability of 2D Silica, Silicide, and Silicene on Pt(111) and Rh(111)
Source: ACS Appl Mater Interfaces. 2024 May 15;16(21):27481–9. doi: 10.1021/acsami.4c05137 (PMC11145594; doi:10.1021/acsami.4c05137)
Supplement: Supplementary file 1 — am4c05137_si_001.pdf [file am4c05137_si_001.pdf]

Supporting Information for

Interface Effects in the Stability of 2D Silica, Silicide and Silicene on Pt(111) and Rh(111)

Matthias Krinninger,[†] *Florian Kraushofer,*[†] *Nils B. Refvik,*[‡] *Monika Blum,*[¶~] *and*

*Barbara A.J. Lechner**[†,§]

† Technical University of Munich, TUM School of Natural Sciences, Department of Chemistry, Functional Nanomaterials Group, Lichtenbergstr. 4, D-85748 Garching, Germany

‡ Department of Physics, University of Alberta, Edmonton, Alberta, T6G 2E1, Canada

¶ Chemical Sciences Division, Lawrence Berkeley National Laboratory, Berkeley, CA 94720, USA

~ Advanced Light Source, Lawrence Berkeley National Laboratory, Berkeley, CA 94720, USA

[§] Institute for Advanced Study, Technical University of Munich, 85748 Garching, Germany

* bajlechner@tum.de

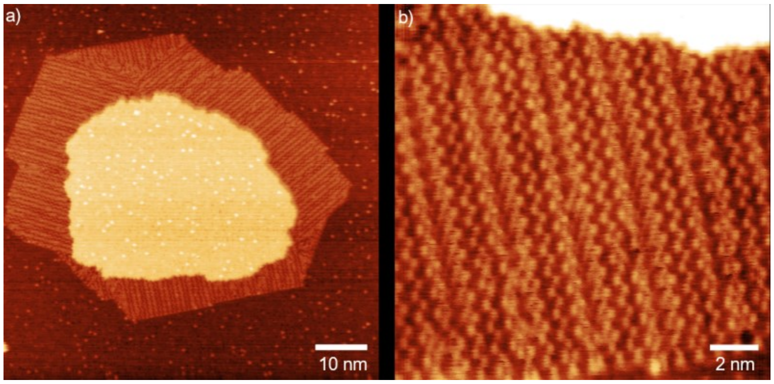

Figure S1. (a) STM image of the $SiO_{2.17}$ "zig-zag" phase[1] on Pt(111), coexisting with the amorphous 2D silica. (b) Zoomed-in STM image of the zig-zag phase in (a). Tunneling parameters: (a) I_t = 0.30 nA, V_b = 0.91 V, 70 × 70 nm^2, (b) I_t = −0.18 nA, V_b = −0.91 V, 15 × 15 nm^2

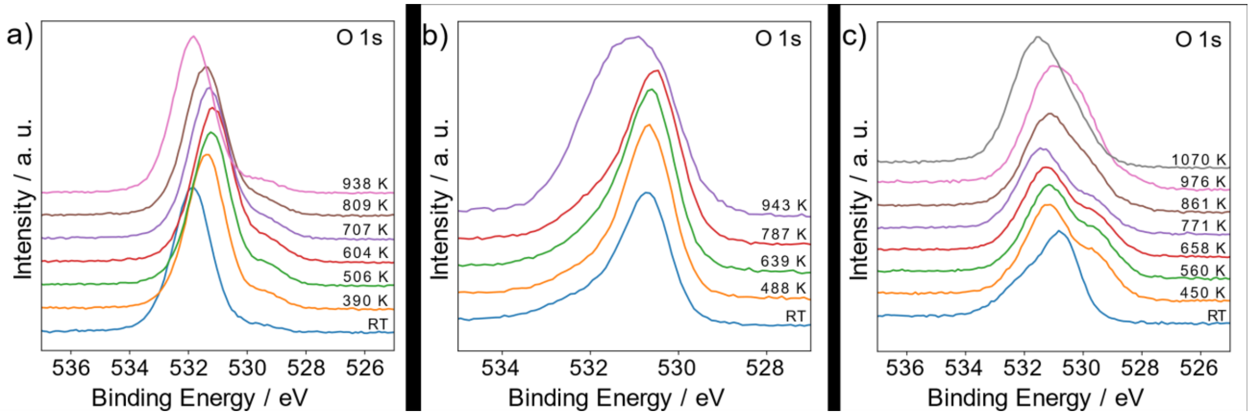

Figure S2. O 1s XPS spectra acquired during film synthesis in 5.0×10^{-6} mbar O_2 for (a) a full monolayer on Rh(111), (b) a full monolayer on Pt(111) and (c) a sub-monolayer film on Pt(111), using an excitation energy of 650 eV.

Table S1. Evolution of binding energies of the Si 2p (excitation energy 300 eV) and O 1s (excitation energy 650 eV) XPS signals and their difference Δ during the 2D SiO_2 film synthesis on Rh(111).

**T / K**	**BE (Si 2p) / eV**	**BE (O 1s) / eV**	**Δ / eV**
RT	102.8	531.9	429.1
390	102.4	531.4	429.0
506	102.2	531.2	429.0
604	102.0	531.1	429.1
707	102.2	531.3	429.1
809	102.7	531.4	428.7
938	102.9	531.8	428.9

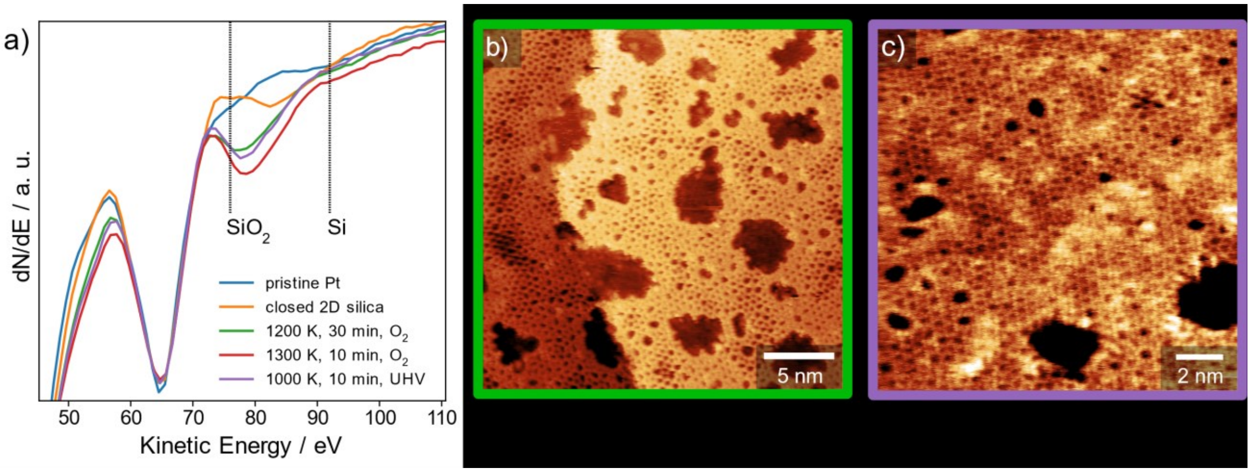

Figure S3. (a) AES spectra (excitation energy 5 keV) of pristine Pt(111) (blue line), fully closed 2D silica on Pt(111) (orange line), the same film after subsequent annealing at T = 1200 K in $p(O_2)$ = 5.0×10^{-6} mbar for 30 minutes (green line), additional annealing at T = 1300 K in $p(O_2)$ = 5.0×10^{-6} mbar for 10 minutes (red line), and additional annealing at T = 1000 K in UHV for 10 minutes (purple line). All spectra are normalized to the Pt signal at 65 eV. As a guide to the eye, the literature values[2] of the kinetic energies of SiO_2 and elemental Si are marked by dashed lines. (b) STM image of the initially closed 2D silica after the first annealing step (corresponding to the green line in (a)), (c) STM image after the last additional annealing step (corresponding to the purple line in (a)).

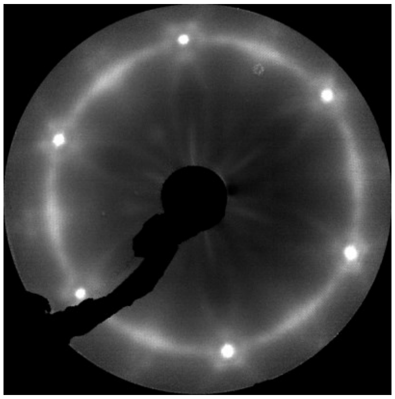

Figure S4. LEED image (103 eV) of fully closed 2D silica on Pt(111), corresponding to the STM images in Figure 1 (d, e) in the main text.

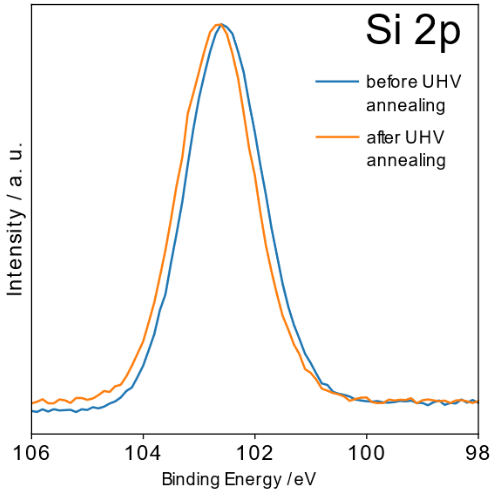

Figure S5. Si 2p XPS spectra of a monolayer 2D silica on Pt(111) before (blue) and after (orange) annealing at 960 K in UHV, measured with an excitation energy of 300 eV.

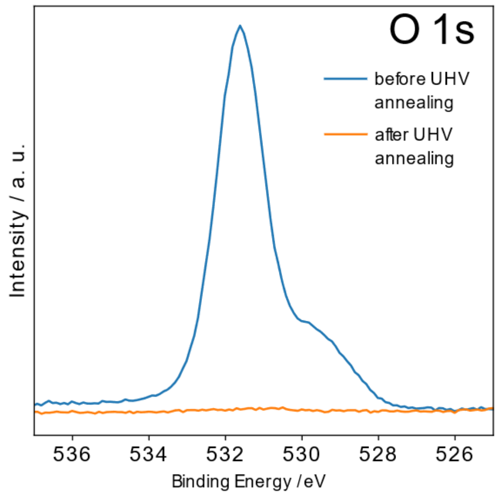

Figure S6. O1s XPS spectra of 2D silica on Rh(111) before (blue) and after (orange) annealing at 946 K in UHV, measured with an excitation energy of 650 eV.

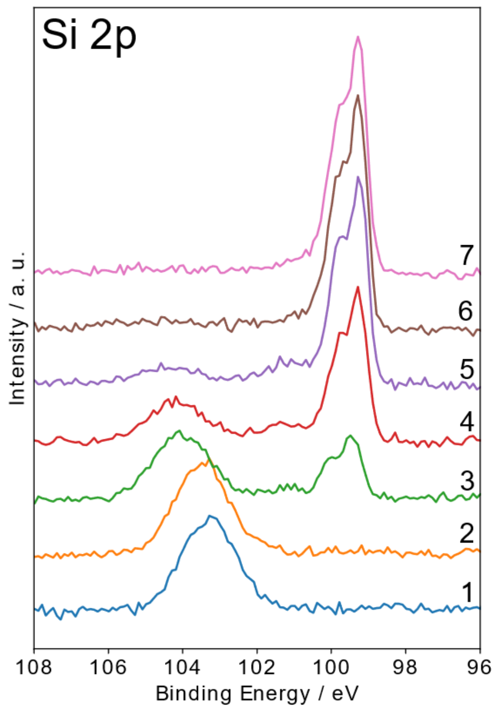

Figure S7. Si 2p XPS spectra (excitation energy 300 eV) of the reduction of the re-oxidized surface silicide on Rh(111) at 665 K. Sweep 1 was recorded while the leak valve that had stabilized a pressure of $p(O_2) = 5\times10^{-6}$ mbar was closed. Full reduction occurs within 6 subsequent sweeps from the time of closing the valve. The time for one sweep is 46 s.

REFERENCES

(1) Kuhness, D.; Yang, H. J.; Klemm, H. W.; Prieto, M.; Peschel, G.; Fuhrich, A.; Menzel, D.; Schmidt, T.; Yu, X.; Shaikhutdinov, S.; Lewandowski, A.; Heyde, M.; Kelemen, A.; Włodarczyk, R.; Usvyat, D.; Schütz, M.; Sauer, J.; Freund, H. J. A Two-Dimensional "Zigzag" Silica Polymorph on a Metal Support. *J. Am. Chem. Soc.* **2018**, *140* (19), 6164–6168.

(2) Davis, L. E.; MacDonald, N. C.; Palmberg, P. W.; Riach, G. E.; Weber, R. E. *Handbook Of Auger Electron Spectroscopy* **1976**, Second Edi.; Physical Electronics Division Perkin Elmer Corporation.
